# Systematic Review of topotecan (Hycamtin) in relapsed small cell lung cancer

**DOI:** 10.1186/1471-2407-10-436

**Published:** 2010-08-17

**Authors:** Rob Riemsma, Jean P Simons, Zahid Bashir, Caroline L Gooch, Jos Kleijnen

**Affiliations:** 1 Kleijnen Systematic Reviews Ltd., York, UK; 2 St. Elisabeth Ziekenhuis, Dept. of Pulmonology, Tilburg, Netherlands; 3 GlaxoSmithKline, UK; 4 School for Public Health and Primary Care (CAPHRI), Maastricht University, Maastricht, Netherlands

## Abstract

**Background:**

To undertake a systematic review of the available data for oral and intravenous topotecan in adults with relapsed small cell lung cancer (SCLC) for whom re-treatment with the first line regimen is not considered appropriate.

**Methods:**

We searched six databases from 1980 up to March 2009 for relevant trials regardless of language or publication status. Relevant studies included any randomised trial of any chemotherapeutic treatment against any comparator in this licensed indication. Where possible we used apposite quantitative methods. Where meta-analysis was considered unsuitable for some or all of the data, we employed a narrative synthesis method. For indirect comparisons we used the method of Bucher et al., where available data allowed it, otherwise we used narrative descriptions.

**Results:**

Seven unique studies met the inclusion criteria, four of which could be used in our analyses. These included one study comparing oral topotecan plus best supportive care (BSC) to BSC alone, one study comparing intravenous topotecan to cyclophosphamide, adriamycin and vincristine (CAV), and two studies comparing oral topotecan with intravenous topotecan. All four studies appear to be well conducted and with low risk of bias.

Oral topotecan plus BSC has advantages over BSC alone in terms of survival (hazard ratio = 0.61; 95% CI, 0.43 to 0.87) and quality of life (EQ-5 D difference: 0.15; 95% CI, 0.05 to 0.25). Intravenous topotecan was at least as effective as CAV in the treatment of patients with recurrent small-cell lung cancer and resulted in improved quality-of-life with respect to several symptoms.

CAV was associated with significantly less grade 4 thrombocytopenia compared with IV topotecan (risk ratio = 5.83; 95% CI, 2.35 to 14.42). Survival (hazard ratio = 0.98; 95% CI, 0.77 to 1.25) and response (pooled risk ratio = 1.04; 95% CI, 0.58 to 1.85) data were similar for the oral and IV topotecan groups. Symptom control was also very similar between the trials and between the oral and IV groups. Toxicity data showed a significant difference in favour of oral topotecan for neutropenia (pooled risk ratio = 0.65; 95% CI, 0.47 to 0.89).

Indirect evidence showed that oral topotecan was at least as good as or better than CAV on all outcomes (survival, response rates, toxicities, and symptoms) that allowed indirect comparisons, with the only exception being grade four thrombocytopenia which occurred less often on CAV treatment.

**Conclusions:**

Concerning topotecan both the oral and intravenous options have similar efficacy, and patient preference may be a decisive factor if the choice would be between the two formulations. The best trial evidence for decision making, because it was tested versus best supportive care, exists for oral topotecan. Indirectly, because we have two head-to-head comparisons of oral versus intravenous topotecan, and one comparison of intravenous topotecan versus CAV in similar patients as in the trial against best supportive care, one might infer that IV topotecan and CAV could also be superior to best supportive care, and that oral topotecan has similar effects to CAV with possibly better symptom control. From the evidence discussed above, it is evident that oral topotecan has similar efficacy to IV topotecan (direct comparison) and CAV (indirect comparison). There is no further evidence base of direct or possible indirect comparisons for other comparators than CAV of either oral or IV topotecan.

## Background

Small cell lung cancer (SCLC) is a type of lung cancer which grows rapidly and spreads quickly to distant sites. Common symptoms of SCLC include weight loss, malaise, bone pain, breathlessness and haemoptysis. SCLC is frequently associated with distinct paraneoplastic syndromes which are not due to direct invasion of adjacent tissues by the cancer or its metastases, for example, neurological or endocrine syndromes.

Lung cancer accounts for around 33,000 deaths per year in England and Wales[1,2]. It is estimated that SCLC constitutes about 10% of the total cases representing about 3,300 new cases per year. Of these, around 24% are classed as limited stage at diagnosis (tumour confined to one side of the chest or to the neck lymph nodes), while the remainder have extensive stage disease (defined as the presence of obvious metastatic disease). The proportion of lung cancer cases of small cell type has been steadily falling over the years and reasons for this are unclear, but it has been attributed to changing smoking habits and a reduction in the tar content of cigarettes.

The prognosis of SCLC is poor; the life expectancy of those with untreated SCLC is about 3.5 months for limited disease and 6 weeks for extensive disease[3]. Prognosis has been linked to performance status and extent of disease, among other factors[4,5].

Current management usually consists of combination chemotherapy regimens. Median survival with such cytotoxic treatment is approximately 14 to 18 months for limited disease and 9 to 12 months for extensive disease. Radiotherapy may be given concurrently with chemotherapy or as part of palliative care. Surgery is only suitable for a small minority of patients with no evidence of local spread or metastasis.

The NICE lung cancer clinical guideline (No. 24) advises that all patients with newly diagnosed SCLC should be offered a platinum-based chemotherapy, and multi-drug regimes[6]. Patients with limited-stage SCLC should be offered thoracic irradiation concurrently with the first or second cycle of chemotherapy, or following completion of chemotherapy if there has been at least a good partial response within the thorax. For patients with extensive disease, thoracic irradiation should be considered following chemotherapy if there has been a complete response at distant sites and at least a good partial response within the thorax. Second-line chemotherapy should be offered to patients at relapse only if their disease responded to first-line chemotherapy.

Topotecan (Hycamtin, GlaxoSmithKline) acts by inhibiting topoisomerase I, an enzyme that is required for DNA replication, leading to cell death. It can be administered either orally or intravenously. Topotecan is indicated as monotherapy for patients with relapsed small cell lung cancer [SCLC] for whom re-treatment with the first-line regimen is not considered appropriate.

Topotecan hard capsules gained a positive opinion from the Committee for Human Medicinal Products (CHMP) on 24 January 2008 *"as monotherapy for the treatment of adult patients with relapsed small cell lung cancer (SCLC) for whom re-treatment with the first line regimen is not considered appropriate." *[7].

The definition of *relapsed *SCLC needs careful consideration and distinction should be made between patients who relapse within 3 months of first line therapy (often called refractory disease) in whom second line chemotherapy or best supportive care (BSC) are the treatment options, and patients who relapse more than 3 months after therapy (relapsed disease, "sensitive" disease) in whom re-treatment with first line therapy is the standard approach. Standard first line chemotherapy has historically been cyclophosphamide, doxorubicin (adriamycin) and vincristine (CAV) though increasingly platinum/etoposide (PE) is the preferred regimen. However, it is also possible other regimens were used as first-line therapy and/or are used as second line therapy in relapsed patients. For example, a fairly recent health technology assessment report from AHRQ in the United States focused on radiotherapy, but also presented results from nine randomised trials which had made 9 different comparisons for second- or subsequent-line treatment of SCLC[8]. Two randomised trials had directly compared chemotherapy with best supportive care for recurrent SCLC[9,10].

Based on the AHRQ report and clinical experience from Europe it seems that there may be a broad set of possible comparators in clinical practice. It would be useful to include an analysis of randomised trials performed in the appropriate patient groups which have used any comparator and consider them for indirect comparisons with topotecan and, if the data allow, possibly as a network meta-analysis[11]. The basic principle of such an approach is that if a randomised trial of A versus B, and another randomised trial of B versus C exist *with the same patient characteristics*, an indirect comparison of A versus C is possible.

Our aim was to undertake a comprehensive systematic review of topotecan and all relevant comparator therapies for the treatment of relapsed SCLC.

## Methods

### Inclusion criteria

We included any randomised trial of any chemotherapeutic treatment against any comparator in patients with SCLC who have relapsed after first line therapy in whom re-treatment with the first line regimen is not considered appropriate.

### Literature searches

We attempted to identify all relevant trials regardless of language or publication status (published, unpublished, in press, and in progress). The search strategies (keywords) were developed specifically for each database (see Additional files 1, 2 and 3). We searched the following databases from 1980 up to September 2008: MEDLINE, EMBASE, CDSR, CENTRAL, DARE, and HTA: Update searches were performed in March 2009.

Furthermore, references in retrieved articles and systematic reviews were checked, and the internet was searched via Google for relevant studies. Also the websites of licensing agencies and HTA agencies were checked. Identified references were downloaded in Reference Manager software for further assessment and handling.

### Methods of trial selection, quality assessment and data extraction

#### Trial selection

Two reviewers independently inspected the abstract of each reference identified by the search and determine the potential relevance of each article. For potentially relevant articles, or in cases of disagreement, the full article was obtained, independently inspected, and inclusion criteria applied. Any disagreements were resolved through discussion.

#### Assessment of methodological quality

We used the Cochrane Collaboration quality assessment checklist[12]. Quality assessment was carried out independently by two reviewers. Any disagreements were resolved by consensus. The results of the quality assessment were used for descriptive purposes to provide an evaluation of the overall quality of the included studies and to provide a transparent method of recommendation for design of any future studies. In addition, we planned if enough data were available from the included studies, to include each of the quality components as explanatory variables in a meta-regression analysis to investigate the association of each of these components with study results as a way of explaining possible heterogeneity. However, this was not possible as we only included 4 studies (See Additional file 4 and the Results section). Based on the findings of the quality assessment, recommendations are made for the conduct of future studies.

#### Data collection

For each study, data were extracted independently by two reviewers. Any disagreements were resolved by consensus.

The following main information was extracted for studies: author, year, country, aim of the study, duration of follow up, description of the participants included in the study, predefined inclusion and/or exclusion criteria, and number of participants recruited/included in the study, details and characteristics of the interventions and control treatments (dosage, length of treatment), number of patients with outcome data per group, reasons for withdrawals and dropouts per group.

Dichotomous data were extracted as the number of individuals with the outcome of interest and the total numbers of individuals in the intervention and control group. Continuous data were extracted as the mean and standard deviation (SD) for the intervention and control group. Survival data were extracted as the hazard ratio and its standard error. Where necessary, we used the formula [upper limit of the 95% CI minus lower limit of the 95% CI] divided by 3.92 to estimate the standard error from the 95% CI, as recommended in the Cochrane Handbook[12].

Finally we extracted the overall conclusion of the study, provided a summary statement of the overall quality of each study, and any additional comments on the study.

#### Data synthesis

Where meta-analysis was considered unsuitable for some or all of the data, we employed a narrative synthesis method. This involved the use of narrative text and tables to summarise data in order to allow the reader to consider outcomes in the light of differences in study designs and potential sources of bias for each of the studies being reviewed. This also involved organizing the studies by (as appropriate) intervention, population, or outcomes assessed, summarizing the results of the studies, summarizing the range and size of the associations these studies report, and describing the most important characteristics of the included studies. A detailed commentary on the major methodological problems or biases that affected the studies is also included, together with a description of how this in our judgement has affected the individual study results.

However, where possible we used the following quantitative methods:

Dichotomous data were analysed by calculating the relative risk (RR) for each trial using the DerSimonian and Laird's method and the corresponding 95% confidence intervals. Continuous data were analysed using the (weighted) mean difference between groups and the corresponding 95% confidence interval. Survival data were analysed by using the hazard ratio (HR) and its standard error (estimated as described above from the 95% CI where not given in the original paper).

We anticipated that systematic differences between studies (heterogeneity) would be likely. Therefore, the random-effects model was used for the calculation of relative risks or weighted mean differences. Heterogeneity was initially assessed by measuring the degree of inconsistency in the studies' results (I^2^). This measure (I^2^) describes the percentage of total variation across studies that is due to heterogeneity rather than the play of chance. The value of I^2 ^lies between 0% and 100%, and a simplified categorization of heterogeneity could be low, moderate, and high to I^2 ^values of 25%, 50%, and 75%.

In the event of important heterogeneity we intended to formally investigate it using meta regression; however, this was not possible because results of only two studies could be pooled.

Statistical analyses were performed using RevMan (version 5) software.

For indirect comparisons we used the method of Bucher et al. (1997)[13], where available data allowed it, otherwise we used narrative descriptions. Bucher's method was implemented using RevMan by performing subgroup analyses, the different subgroups being defined by the different comparisons being made. For the particular case of two subgroups (two comparisons; three interventions) the difference between the subgroups can be estimated, and the statistical significance determined, using Bucher's method. In this review, one subgroup would be the 'oral topotecan versus IV topotecan' trials, and the other subgroup the 'CAV versus IV topotecan' trial. The difference between the summary effects in the two subgroups provided an estimate of the desired comparison, 'oral topotecan versus CAV'. The test was performed using the test for differences between subgroups, as implemented in RevMan version 5.

##### Sub-group analysis

Where sufficient data were available we planned to present subgroup analyses to investigate whether the efficacy differs according to:

• age group

• gender

• performance status

• prior therapy

• extent of disease

• presence of any liver metastases

• patients for whom an IV chemotherapy is unsuitable

• patients with serious pre-existing cardiovascular or neurological conditions, for whom treatment with an anthracycline-based regimen would not be clinically appropriate

• time to relapse/progression

## Results

**Figure 1:**
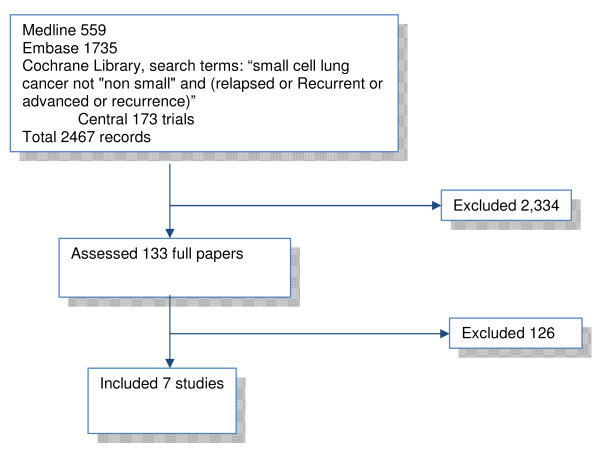
**Search strategy**.

Our searches of the databases for topotecan trials yielded 2,467 titles and abstracts; of these we ordered and assessed 133 full papers for possible inclusion, and from these 133 papers, 7 unique studies met the inclusion criteria (figure 1).

Of the 133 full papers which we assessed for possible indirect comparisons, 66% were excluded because they reported on patients who did not previously receive chemotherapy, 14% were not randomised trials, 9% did not have any usable comparator (e.g. studies randomising to different dosages of the same intervention), and 6% treated patients who were in complete remission.

Thus, we identified seven studies (5%) for our analyses. These included one study comparing oral topotecan plus best supportive care to best supportive care alone (O'Brien 2006[9]), one study comparing intravenous topotecan to CAV (von Pawel 1999[14]), two studies comparing oral topotecan with intravenous topotecan (von Pawel 2001[15], Eckardt 2007[16]), one study comparing etoposide/cisplatinum with bis-chloro-ethynylnitrosourea/thiotepa/vincristine/cyclophosphamide (O'Bryan et al 1990[17]), one study comparing CCNU/cyclophosphamide/etoposide with CAV (Gervais et al. 2007[18]) and one study comparing topotecan with amrubicin (Inoue et al. 2008[19]).

The study by O'Brien et al addresses the question: "What is the evidence that chemotherapy can have beneficial effects in these patients?" This question is best answered by a randomised trial of chemotherapy plus best supportive care versus best supportive care alone.

The three studies by Von Pawel 1999 & 2001 and Eckardt 2007 address the question "What is the evidence for the comparative effects of different chemotherapeutic treatments in these patients?" This question is best answered by a randomised trial with head-to-head comparisons. Patients in these three trials had recurrence at least 60 days, 3 months and 90 days after the end of first-line chemotherapy, respectively. For these patients re-treatment with first line therapy is the standard approach. The licence indication for topotecan states that it is for patients "*for whom re-treatment with the first line regimen is not considered appropriate"*. The fact that these patients were selected for topotecan trials led us to assume they were unsuitable for retreatment with the first line regimen, and therefore fulfilled our inclusion criteria.

The study by Gervais et al. 2007 only provided a methods description of the trial and no results are reported; the study by O'Bryan et al. 1990 provided no comparator that is common to any of the other studies; while the study by Inoue et al. 2008 uses a lower dose (1.0 mg/m^2^) than usual for intravenous topotecan. Therefore we did not include these three studies for our analyses.

**Figure 2:**
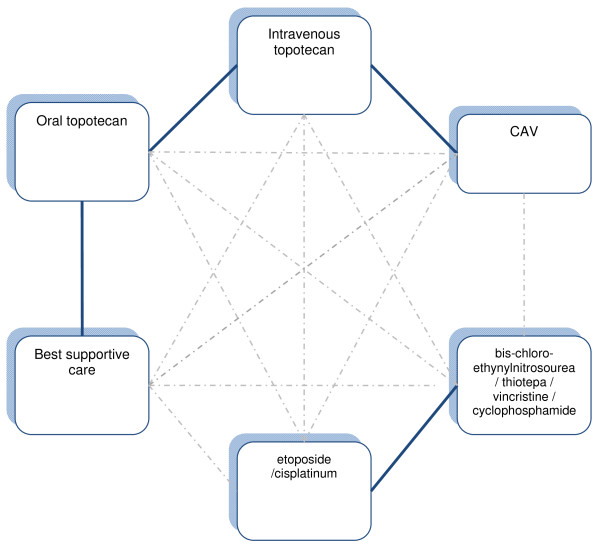
**Network of Available Comparisons**.

This left the network of available comparisons as presented in figure 2.

The totality of this evidence addresses the main questions about oral topotecan for second-line treatment in relapsed patients with resistant or sensitive disease. Based on our searches, it appears that only for oral topotecan randomised trials addressing both questions exist. For CAV and for intravenous topotecan we did not find a randomised study assessing their efficacy versus best supportive care in these patients. Actually, for second-line treatment in these patients we found no other randomised trials of CAV.

The quality of these studies is presented in Additional file 4. Given that blinding of investigators (physicians) and patients would be very difficult with these regiments, there is likely to be some risk of bias resulting from this. However, in general all 4 studies appear to be well conducted and with low risk of bias.

### Direct comparisons

The main characteristics of the included studies are presented in table 1.

**Table 1:** Main characteristics of included trials

	O'Brien 2006	Von Pawel 1999	Von Pawel 2001	Eckardt 2007
**Comparison**	**Oral topotecan **(2.3 mg/m^2^/d on days 1-5 every 21 days) vs **BSC**	**IV topotecan **(1.5 to 2 mg/m^2^/d (as 30-minute infusion) on days 1-5 every 21 days) vs **CAV **(cyclophosphamide 1,000 mg/m^2 ^(max 2,000 mg), doxorubicin 45 mg/m^2 ^(max 100 mg), and vincristine 2 mg, administered on day 1 of each course)	**Oral **(2.3 mg/m^2^/d) vs **IV topotecan **(1.5 mg/m^2^/d (as 30-minute infusion) on days 1-5 every 21 days)	**Oral **(2.3 mg/m^2^/d) vs **IV topotecan **(1.5 mg/m^2^/d (as 30-minute infusion) on days 1-5 every 3 weeks)

**Design**	Multinational, multicentre, open-label, phase III RCT	Multinational, multicentrel open-label RCT	Multinational, multicentre, open-label, phase II RCT	Multinational, multicentre, open-label, phase III RCT

**N**	141	211	106	304

**Inclusion criteria**	Documented relapse of limited or extensive SCLC at least 45 days after the cessation of first-line chemotherapy. Not considered suitable for further intravenous chemotherapy. Received one prior chemotherapy regimen only. Documented partial or complete response to first-line therapy.	Patients with documented progressive, limited or extensive SCLC that had recurred at least 60 days after the end of first-line chemotherapy	Patients with limited or extensive SCLC that had recurred at least 3 months after the end of first-line chemotherapy. Received one prior chemotherapy regimen only.	Patients with limited or extensive SCLC and documented complete or partial response to first-line therapy with disease recurrence after ≥90 days. Received one prior chemotherapy regimen only

**Mean age (range)**	59.2 (37-79) years	Not reported	59.0 (35-79) years	62.2 (35-82) years

**% extensive disease**	65%	84%	72%	68%

**Performance status (% 0/1/2)**	10/60/29%	18/61/21%	26/52/22%	27/60/13%

**Previous chemotherapy**	Not reported	Platinum/etoposide: 48% CAV: 1%	Not reported	cisplatinum or carboplatinum + etoposide;
		Both platinum/etoposide + CAV: 14% Cyclophosphamide/doxorubicin/etoposide : 17%		vincristine + cisplatinum or carboplatinum + etoposide;
		Vincristine/platinum/etoposide: 5% Other regimens: 15%		cyclophosphamide +epirubicine + cisplatinum or carboplatinum + etoposide

**Previous surgery**	27%	21%	Not reported	Not reported

**Previous radiotherapy**	51%	59%	Not reported	Not reported

**Previous immunotherapy**	3%	1%	Not reported	Not reported

**Liver metastases**	24%	40%	31%	29%

The most important effectiveness outcomes are summarised in table 2 and table 3 shows adverse events. None of the comparisons concerning the effectiveness outcomes showed a statistically significant advantage for either BSC or CAV. Several outcomes showed statistically significant advantages for topotecan, these are presented in bold.

**Table 2:** Main outcomes

	O'Brien 2006	Von Pawel 1999	Von Pawel 2001	Eckardt 2007
	**Oral topotecan vs BSC**	**IV topotecan vs CAV**	**Oral vs IV topotecan**	**Oral vs IV topotecan**

**Overall survival (median)**	**25.9 (18.3 to 31.6) weeks vs 13.9 (11.1 to 18.6) weeks**	25 (0.4 to 90.7) weeks vs 24.7 (1.3 to 101.3) weeks	32.3 (0.4-69.1) weeks vs 25.1 (0.6-65.1) weeks	33.0 (29.1 to 42.4) weeks vs 35.0 (31.0 to 37.4) weeks

**Overall survival [hazard ratio]**	**0.61 (0.43 to 0.87)**	1.04 (0.78 to 1.40)†	Risk ratio 0.90 (0.55 to 1.47)	0.98 (0.77 to 1.25)

**Progression free survival**				

**Time to progression**	16.3 (12.9 to 20.0) weeks*	13.3 (0.4 to 55.1) weeks vs 12.3 (0.1 to 75.3) weeks	15 weeks vs 13 weeks	

**Response rate [risk ratio]**		1.33 (0.79 to 2.25)$	1.56 (0.69 to 3.50)$	0.84 (0.53 to 1.31)$

**Response rate**	7%*	24.3% (16.2 to 32.4) vs18.3% (10.8 to 25.7); difference 6.0% (-6 to 18)	23% vs 15% difference 8.3% (-6.6% to 23.1%)	18.3% (12.2 to 24.4) vs 21.9% (15.3 to 28.5%) difference - 3.6% (-12.6% to 5.5%)

**Complete response**		0% vs 1%		

**Response duration**		14.4 (9.4 to 50.1) weeks vs 15.3 (8.6 to 69.9) weeks	18 weeks vs 14 weeks	18.3 weeks vs 25.4 weeks

**Stable disease**	44%*	19.6% vs 11.5%; OR 1.87 (0.87 to 4.03)$		

**Quality of Life**	**Difference in rate of change EQ-5 D 0.15 (0.05 to 0.25) in favour of topotecan**			FACT-L total score (change from baseline) -5.07 (-7.49 to -2.65) vs -5.67 (-8.05 to 3.28); difference 0.59 (-2.38 to 3.57)#

Effects in bold show advantage for topotecan; effects in italics show advantage for best supportive care or CAV (none were found); numbers between brackets are 95% confidence intervals

*only provided for topotecan group

vs = versus

$ = own calculation from 2 by 2 data

# = data supplied by GlaxoSmithKline

† = data from EMEA: EPAR - Hycamtin-H-123-II-34; see:

http://www.ema.europa.eu/docs/en_GB/document_library/EPAR_-_Scientific_Discussion_-_Variation/human/000123/WC500051543.pdf

BSC: best supportive care

**Table 3:** Improvement in symptom control

	O'Brien 2006	Von Pawel 1999	Von Pawel 2001	Eckardt 2007
***Improvement in:***	**Oral topotecan vs BSC**	**IV topotecan vs** **CAV**	**Oral vs IV** **topotecan**	**Oral vs IV topotecan#**

**Shortness of breath**	**OR 2.18 (1.09 to 4.38)**	**27.9% vs 6.6% P = 0.002**	13.8% vs 27.3% P = 0.20$	0.009 (-0.21 to 0.23) vs 0.08 (-0.29 to 0.14) difference 0.08 (-0.22 to 0.39)†

**Cough**	OR 1.35 (0.68 to 2.66)	24.6% vs 14.8% P = 0.16	16.1% vs 22.2% P = 0.53$	0.14 (-0.07 to 0.36) vs 0.27 (0.07 to 0.46) difference -0.13 (-0.41 to 0.16)†

**Chest pain**	OR 2.07 (1.00 to 4.28)	25.0% vs 17.1% P = 0.371	42.1% vs 31.8% P = 0.50$	-0.18 (-0.40 to 0.03) vs 0.06 (-0.11 to 0.23) difference -0.24 (-0.52 to 0.03)†

**Coughing up blood**	OR 1.95 (0.46 to 8.27)	26.7% vs 33.3% P = 0.706	33.3% vs 40.0% P = 0.84$	

**Loss of appetite**	OR 1.02 (0.57 to 1.84)	**32.1% vs 15.8% P = 0.042**	18.5% vs 31.0% P = 0.28$	

**Interference with sleep**	**OR 2.16 (1.15 to 4.06)**	33.3% vs 18.9% P = 0.085	32.0% vs 26.6% P = 0.85$	

**Hoarseness**	OR 1.35 (0.63 to 2.87)	**32.5% vs 13.2% P = 0.043**	35.7% vs 37.5% P = 0.91$	

**Fatigue**	**OR 2.29 (1.25 to 4.19)**	**22.9% vs 9.2%** **P = 0.032**	21.2% vs 16.7% P = 0.63$	

**Interference with** **daily activities**	OR 1.70 (0.95 to 3.03)	**26.9% vs 11.1% P = 0.023**	25.8% vs 22.2% P = 0.73$	

**Nausea**				-0.42 (-0.60 to -0.24) vs -0.40 (-0.60 to -0.21) difference -0.02 (-0.28 to 0.24)†

**Pain**				-0.08 (-0.28 to 0.12) vs -0.40 (-0.60 to -0.21) difference 0.09 (-0.22 to 0.40)†

Effects in bold show advantage for topotecan; effects in italics show advantage for best supportive care or CAV (none were found); numbers between brackets are 95% confidence intervals; BSC best supportive care; OR = odds ratio; vs versus; $ = own calculation from 2 by 2 data; † = data supplied by GlaxoSmithKline; # a positive change indicates improvement

#### Oral topotecan versus best supportive care

Table 2 shows that for important effectiveness outcomes oral topotecan plus best supportive care has advantages over best supportive care alone. O'Brien et al. conclude that "chemotherapy with oral topotecan is associated with prolongation of survival and quality of life benefit in patients with relapsed small-cell lung cancer.

#### Intravenous topotecan versus CAV

The study by von Pawel et al 1999 concludes that (intravenous) topotecan was at least as effective as CAV in the treatment of patients with recurrent small-cell lung cancer and resulted in improved control of several symptoms.

#### Oral topotecan versus intravenous topotecan

Two studies assessed oral versus intravenous topotecan, and these have included similar patients and used the same interventions. A number of the outcomes allow meta-analysis, and others can be compared narratively.

**Figure 3:**
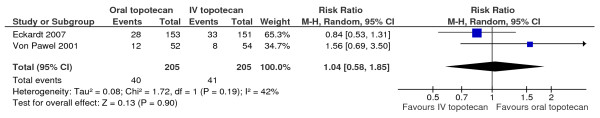
**Overall response rates**.

Combined overall response rates show no differences between oral and intravenous topotecan, although the I^2 ^statistic shows moderate heterogeneity (figure 3). The cause is not immediately apparent, but von Pawel 2001 has a limited number of event rates and a wide 95% confidence interval.

Survival data were similar in both trials and similar for the oral and IV topotecan groups. In the Eckardt 2007 trial, median survival time was 33.0 weeks (95% CI, 29.1 to 42.4 weeks) in the oral group and 35.0 weeks (95% CI, 31.0 to 37.4 weeks) in the IV group, with data censored for 13.7% and 10.6% of patients in the respective groups. Cox proportional hazards regression showed no difference between the two groups (hazard ratio = 0.98; 95% CI, 0.77 to 1.25). At 1 year, the survival rate was 33% after treatment with oral topotecan and 29% after treatment with IV topotecan; the 2-year survival rates were 12% for oral topotecan and 7% for IV topotecan. In the von Pawel 2001 trial, median survival was 32 weeks in the oral group and 25 weeks in the IV group. The risk ratio (oral:IV) for survival was 0.84 (95% CI, 0.53 to 1.32), and accounted for prognostic factors (response and duration of response to previous therapy, sex, presence of renal impairment at baseline, performance status, presence of baseline liver metastases, extent of disease, previous radiotherapy, and maximum tumor diameter) it was 0.90 (95% CI, 0.55 to 1.47).

Symptom control was also very similar between the trials and between the oral and IV groups.

Toxicity data show a few differences, with neutropenia (pooled risk ratio = 0.65 (95% CI, 0.47 to 0.89) and leucopenia (pooled risk ratio = 0.80 (95% CI, 0.56 to 1.14) showing a tendency in favour of oral topotecan, which for neutropenia is significant in both studies; and with tendencies towards better effects for IV topotecan for thrombocytopenia (pooled risk ratio = 1.42 (95% CI, 0.99 to 2.03) and anaemia (pooled risk ratio = 1.66 (95% CI, 0.61 to 4.52).

### Indirect comparisons

In addition to these direct comparisons, it was possible to perform indirect comparisons for oral topotecan versus CAV (via intravenous topotecan) and intravenous topotecan versus best supportive care (via oral topotecan). The study characteristics (table 1) show that normal assumptions for making indirect comparisons between oral topotecan and CAV are fulfilled: all trials included similar patients, the IV topotecan regimens were similar, follow up was similar and a number of the same outcomes are available.

#### Survival time

In the Eckardt 2007 trial, median survival time was 33.0 weeks (95% CI, 29.1 to 42.4 weeks) in the oral group and 35.0 weeks (95% CI, 31.0 to 37.4 weeks) in the IV group. In the Von Pawel 2001 trial, median survival was 32 weeks in the oral group and 25 weeks in the IV group. In the Von Pawel 1999 trial, median survival was 25 weeks for the IV topotecan group, and 24.7 weeks for CAV. Survival is therefore very similar in all trials, allowing the conclusion that survival for oral topotecan is not worse than survival on CAV.

The calculated hazard ratio between oral topotecan and CAV (using Bucher's method) is HR = 1.02 (95% CI: 0.70, 1.49) and between IV topotecan and BSC: HR = 0.62 (95% CI: 0.41, 0.95).

#### Overall response rates

O'Brien et al. 2007 only reported response rates for patients treated with topotecan. Therefore, an indirect comparison of IV topotecan versus BSC for response can not be performed.

**Figure 4:**
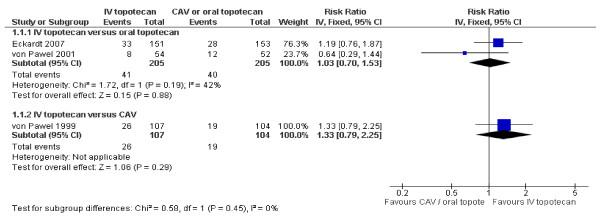
Overall response rates*. * The test for subgroup differences is only possible using the fixed effect model in RevMan. The difference in relative risks between figure 3 and 4 is due to the fact that the random effects model gives relatively more weight to smaller trials.

The figure for overall response rates below (figure 4) shows us that the confidence intervals almost completely overlap, demonstrating that similar response rates are found between oral topotecan and CAV. The p-value for subgroup differences (and thus for the indirect comparison) is 0.45. The calculated risk ratio between oral topotecan and CAV (using Bucher's method) is RR = 1.29 (95% CI: 0.67, 2.50), indicating a non-significant advantage for oral topotecan.

#### Toxicity

O'Brien et al. 2007 only reported toxicity data for patients treated with topotecan. Therefore, an indirect comparison of IV topotecan versus BSC for toxicity can not be performed.

In table 4 the risk ratios for four grade 4 toxicities are reported. As can be seen from the direct comparisons, IV topotecan was associated with significantly more grade 4 thrombocytopenia compared with CAV; and IV topotecan was associated with significantly more grade 4 neutropenia compared with oral topotecan. The indirect comparisons, using Bucher's method, showed that CAV was associated with significantly more grade 4 neutropenia compared with oral topotecan; while oral topotecan was associated with significantly more grade 4 thrombocytopenia compared with CAV.

**Table 4:** Risk ratios for toxicity results

	**IV topotecan vs CAV**^**1**^	**IV vs Oral topotecan**^**2**^	**Oral topotecan vs CAV**^**3**^
**Grade 4 neutropenia**	0.98 (0.82 to 1.17)	1.47 (1.22 to 1.78)	0.67 (0.51 to 0.87)

**Grade 4 thrombocytopenia**	5.83 (2.35, 14.42)	0.70 (0.49 to 1.01)	8.41 (3.17 to 22.35)

**Grade 4 anaemia**	1.46 (0.25 to 8.54)	0.60 (0.22 to 1.65)	2.46 (0.33 to 18.53)

1- Direct comparison (Von Pawel 1999); 2- Direct comparison (Von Pawel 2001 & Eckardt 2007); 3- Indirect comparison (Von Pawel 1999/Von Pawel 2001 & Eckardt 2007).

#### Symptom control

Unfortunately, data about symptoms were not presented in similar ways to allow indirect comparisons. However, the study comparing IV topotecan with CAV reported improved control of several symptoms, whereas the studies of oral versus IV topotecan reported similar effects on symptoms. This allows at least speculation that oral topotecan would also have better symptom control than CAV.

#### Conclusions from indirect comparisons

Overall, oral topotecan was at least as good as or better than CAV on all outcomes (survival, response rates, toxicities, and symptoms) that allowed indirect comparisons, with the only exception being grade four thrombocytopenia which occurred less often on CAV treatment.

## Discussion

Topotecan is indicated as monotherapy for patients with relapsed small cell lung cancer for whom re-treatment with the first-line regimen is not considered appropriate. This indication has an important nuance that deserves further qualification: when is re-treatment with first line regimen not considered appropriate?

The included studies operationalised this issue as follows: the O'Brien study accepted patients with recurrence less than 60 days after the end of first-line chemotherapy, but 70% of patients had recurrence more than 60 days after the end of first-line chemotherapy. Von Pawel's two studies included patients with recurrence at least 60 days after the end of first-line chemotherapy, whereas the Eckardt study included patients with disease recurrence after ≥90 days. Data from these trials, but also from various phase II studies confirm that patients have a better prognosis with a longer interval before recurrence, but the O'Brien trial showed that even patients with poorer prognosis due to shorter interval before recurrence still have survival benefit from oral topotecan (median survival 23.3 weeks; 95% CI 10.7 to 30.9) compared with best supportive care (13.2 weeks; 95% CI 7.0 to 21.0). The NICE lung cancer clinical guideline, which states that second-line chemotherapy should be offered to patients at relapse only if their disease responded to first-line chemotherapy, does not quantify a time frame until recurrence, and states that second-line treatment should be discussed on an individual basis.

The trials show clinical disagreement about the period between 60 and 90 days, but there seems to be general agreement that from 90 days onwards re-treatment with first line chemotherapy should be considered. In addition to the issue of what is the exact cut-off in days after first-line treatment when recurrence appeared, there are other issues that play a role. The O'Brien study addresses this issue in some detail and gives a number of reasons why retreatment with first-line therapy might not be appropriate, including patient preference (table 5)[9].

**Table 5:** Study 478: Potential reasons for not being suitable for repeat first line treatment

Reasons	Relevance to study 478 population
Short time to progression	54% of patients in the trial had a treatment free interval (TFI) of ≤90 days

Residual toxicity to first line regimen	13% of patients in the best supportive care + topotecan arm and 10% in the best supportive care arm had residual toxicity

Patient preference not to receive further intravenous chemotherapy	O'Brien et al report that some patients in the study with sensitive SCLC refuse further intravenous chemotherapy because of the risk of toxicity or become unsuitable for standard chemotherapy because of co-morbidities

This concurs with the NICE guidance that second-line treatment should be discussed on an individual basis, and with the findings of Liu et al 1997[20].

Three out of the four included studies (O'Brien 2006, von Pawel 1999 and von Pawel 2001) reported the presence of liver metastases as a factor that negatively influenced the outcomes. Von Pawel 2001 reported that patients with baseline liver metastases had a risk that was 1.9 times that of patients without for time to progression. Von Pawel 1999, in a logistic regression model evaluating the effects of baseline characteristics identified presence of baseline liver metastases and sex as the only significant factors of response (*P = *.043 and *P = *.008, respectively). O'Brien 2006 found a more favourable point estimate for the effect of topotecan in patients without liver metastases, although the confidence intervals overlapped. However, data in patients with liver metastases are very limited and none of the authors suggested that patients with liver metastases should be considered differently.

A recent systematic review arrived at largely similar conclusions as our review[21]. Cheng et al 2007 reported that the evidence for the clinical benefit of second-line chemotherapy in the treatment of patients with relapsed SCLC is limited. The selection of patients for treatment with second-line therapy should be dependent on the treatment-free interval, the extent of response to first-line therapy, residual toxicity from first-line therapy, and the performance status of the patient. They go on to conclude that there is insufficient evidence to recommend a specific chemotherapy regimen. Nevertheless, in the opinion of the lung cancer disease site group, patients who relapse three or more months after having completed first-line chemotherapy may benefit from re-treatment with the same regimen that induced their initial response. This would generally mean re-treatment with etoposide and cisplatin. Alternative regimens may include CAV or carboplatin and etoposide. Topotecan is a possible alternative for patients who initially respond to chemotherapy and who have response duration of 45 days or longer. Topotecan may be administered orally or intravenously. Available evidence has not yet established a superior mode of administration, and each has different benefits and toxicities. Oral administration is associated with a higher incidence of grade 3/4 diarrhoea, whereas IV administration may result in a higher frequency of grade 3/4 neutropenia. The final conclusion from Cheng et al 2007 is that there is currently no standard second-line chemotherapy regimen for patients who fail to respond to or who relapse shortly after first-line therapy. Clinical trials are needed to determine the optimal treatment regimen.

Through our searches we found three ongoing studies. The first study is a randomized phase II study, of topotecan versus amrubicin in patients with relapsed SCLC following first-line chemotherapy, results of which were presented by Jotte et al. at the World Lung Cancer Congress 2009 (NCT00319969). Jotte et al. reported a response rate of 44% for amrubicin versus 11.5% for topotecan, with median overall survival of 9.3 versus 7.7 months in favour of amrubicin. The second study was a randomised Phase III study of topotecan versus amrubicin as second line therapy in 620 patients with SCLC (NCT00547651). This study completed accrual in 2010 and is expected to be completed in March 2011. The third study is a comparison of picoplatin (a platinum-based chemotherapy) + BSC versus BSC alone in patients with relapsed SCLC (NCT00465491). The expected completion date is May 2009, but as far as we know results have not yet been published.

So what are the treatment options in patients who relapse after first-line treatment? In addition to topotecan, best supportive care is one, and also CAV. There is a paucity of randomised trials in this group of patients; and most trials (four) have actually evaluated topotecan. CAV was the comparator in one of these topotecan studies. For CAV and for intravenous topotecan we did not find a randomised study assessing their effects versus best supportive care in these patients. Actually, for second-line treatment in patients who relapsed after first-line treatment, we found no other randomised trials of CAV.

Concerning topotecan both the oral and intravenous options have similar effects, and patient preference may be a decisive factor if the choice would be between the two formulations. The best trial evidence for decision making, because it was tested versus best supportive care, exists for oral topotecan. Indirectly, because we have two head-to-head comparisons of oral versus intravenous topotecan, and one comparison of intravenous topotecan versus CAV in similar patients as in the trial against best supportive care, one might infer that IV topotecan and CAV could also be superior to best supportive care, and that oral topotecan has similar effects as CAV with possibly better symptom control. So for patients suitable for additional treatment, topotecan or CAV would be superior treatment options when compared with best supportive care. For patients not willing or unable to have intravenous treatment, oral topotecan is the only choice with evidence of efficacy from randomised trials. According to the study by Liu et al 1997, patients with incurable cancer have a clear preference for oral chemotherapy, but are generally not willing to sacrifice efficacy for their preference. Almost 40% of patients did not want to make final treatment decisions themselves. From the evidence discussed above, it is evident that oral topotecan has similar efficacy to IV topotecan (direct comparison) and CAV (indirect comparison). There is no further evidence base of direct or possible indirect comparisons for other comparators than CAV of either oral or IV topotecan.

## Conclusion

There is high unmet medical need to offer further active treatment in relapsed SCLC and therefore improve outcomes in patients for whom currently available IV second line chemotherapy is not considered an option and therefore whose only treatment option is currently best supporting care (BSC). Topotecan is indicated for the treatment of patients with relapsed small cell lung cancer (SCLC) for whom re-treatment with the first line regimen is not considered appropriate. When compared with BSC alone, oral topotecan combined with BSC extends overall survival, disease-free survival and positively improves quality-of-life across a number of symptoms in SCLC patients who have relapsed following first line therapy.

## Competing interests

The project was funded by GlaxoSmithKline. ZB and CLG both are employed by GlaxoSmithKline, UK. RR, JPS and JK have no other conflicts of interest. Furthermore, RR, JS and JK had ultimate editorial control of the manuscript.

## Authors' contributions

RR (Senior Research Fellow) assessed abstracts and titles for inclusion and exclusion, conducted the systematic review, and contributed to writing and editing the report. JPS (Clinical Expert) advised on clinical matters and the interpretation of the data and contributed to the writing of the protocol and report. ZB (Medical Advisor) advised on clinical matters and the interpretation of the data and contributed to the writing of the protocol and report. CLG (Scientific Advisor Oncology) advised on clinical matters and the interpretation of the data and contributed to the writing of the protocol and report. JK (Director) provided overall project management, wrote the protocol, assessed abstracts and titles for inclusion and exclusion, conducted the systematic review, and contributed to writing and editing the report. All authors read and approved the final manuscript.

## Pre-publication history

The pre-publication history for this paper can be accessed here:

http://www.biomedcentral.com/1471-2407/10/436/prepub

## Supplementary Material

Additional file 1 **Ovid MEDLINE(R) 1950 to August Week 4 2008 Search date 5 September 2008**. Click here for file

Additional file 2 **Ovid MEDLINE(R) In-Process & Other Non-Indexed Citations September 04, 2008 Search date 5 Sept 2008**. Click here for file

Additional file 3 **EMBASE - 1974 to date (EMZZ) search date 5 September 2008**. Click here for file

Additional file 4 **Quality assessment**. Click here for file
