# Supplementary material for: Systematic Review of topotecan (Hycamtin) in relapsed small cell lung cancer
Source: BMC Cancer. 2010 Aug 17;10:436. doi: 10.1186/1471-2407-10-436 (PMC2931489; doi:10.1186/1471-2407-10-436)
Supplement: Additional file 1 — Ovid MEDLINE(R) 1950 to August Week 4 2008 Search date 5 September 2008. [file 1471-2407-10-436-S1.PDF]

Searches		Results
1	exp Carcinoma, Small Cell/	15753
2	small cell lung cancer.mp [mp=title, original title, abstract, name of substance word, subject heading word]	19387
3	1 or 2	29825
4	((Carcinoma, Small Cell or small cell lung cancer) not not-small).m_titl	3677
5	advanced.mp.	158710
6	exp Recurrence/	126568
7	relaps$.mp [mp=title, original title, abstract, name of substance word, subject heading word]	82430
8	second line.mp. [mp=title, original title, abstract, name of substance word, subject heading word]	6401
9	recurren$.mp [mp=title, original title, abstract, name of substance word, subject heading word]	344355
10	5 or 6 or 7 or 8 or 9	540133
11	4 and 10	723
12	Limit 11 to “therapy (sensitivity)”	553
