# Supplementary material for: Systematic Review of topotecan (Hycamtin) in relapsed small cell lung cancer
Source: BMC Cancer. 2010 Aug 17;10:436. doi: 10.1186/1471-2407-10-436 (PMC2931489; doi:10.1186/1471-2407-10-436)
Supplement: Additional file 2 — Ovid MEDLINE(R) In-Process & Other Non-Indexed Citations September 04, 2008 Search date 5 Sept 2008. [file 1471-2407-10-436-S2.PDF]

Searches		Results
1	exp Carcinoma, Small Cell/	0
2	small cell lung cancer.mp. [mp=title, original title, abstract, name of substance word, subject heading word]	1650
3	1 OR 2	1650
4	((Carcinoma, Small Cell or small cell lung cancer) not non-small).m_titl.	160
5	advanced.mp.	12011
6	exp Recurrence/	0
7	relaps$.mp. [mp=title, original title, abstract, name of substance word, subject heading word]	4874
8	second line.mp. [mp=title, original title, abstract, name of substance word, subject heading word]	652
9	recurren$.mp. [mp=title, original title, abstract, name of substance word, subject heading word]	15519
10	5 or 6 or 7 or 8 or 9	31079
11	4 and 10	43
12	limit 11 to "therapy (sensitivity)"	6
