# Supplementary material for: Systematic Review of topotecan (Hycamtin) in relapsed small cell lung cancer
Source: BMC Cancer. 2010 Aug 17;10:436. doi: 10.1186/1471-2407-10-436 (PMC2931489; doi:10.1186/1471-2407-10-436)
Supplement: Additional file 3 — EMBASE - 1974 to date (EMZZ) search date 5 September 2008. [file 1471-2407-10-436-S3.PDF]

No.	Search term	Results
CP	[Clipboard]	0
1	small ADJ cell ADJ lung ADJ cancer	19026
2	Lung-Small-Cell-Cancer#.DE.	9152
3	1 OR 2	23375
4	(non ADJ small).TI.	11046
5	3 NOT 4	13478
6	advanced	168690
7	recurren$	272261
8	second ADJ line	6370
9	relaps$	85416
10	Cancer-Recurrence#.DE.	47782
11	6 OR 7 OR 8 OR 9 OR 10	490536
12	5 AND 11	2766
13	Randomized-Controlled-Trial#.DE.	162847
14	random$	429312
15	phase ADJ III	11997
17	phase ADJ three	374
18	Clinical-Trial#.DE.	526802
19	Controlled-Study#.DE.	2758146
20	Randomization#.W..DE.	26095
21	Prospective-Study#.DE.	76662
22	Follow-Up#.DE.	266712
23	13 OR 14 OR 15 OR 17 OR 18 OR 19 OR 20 OR 21 OR 22	3303050
24	12 AND 23	1752
25	HUMAN=YES	7895713
26	24 AND 25	1735
