# Supplementary material for: Systematic Review of topotecan (Hycamtin) in relapsed small cell lung cancer
Source: BMC Cancer. 2010 Aug 17;10:436. doi: 10.1186/1471-2407-10-436 (PMC2931489; doi:10.1186/1471-2407-10-436)
Supplement: Additional file 4 — Quality assessment. [file 1471-2407-10-436-S4.PDF]

Domain	Von Pawel 2001	Von Pawel 1999	Eckardt 2007	O'Brien 2006
Was the allocation sequence adequately generated?	Unclear	Yes	Yes	Yes
Was allocation adequately concealed?	Unclear	Unclear	Yes	Yes
Was knowledge of the allocated intervention adequately prevented during the study?	No	No	No	No
Were incomplete outcome data adequately addressed?	Yes	No	No	Unclear
Are reports of the study free of suggestion of selective outcome reporting?	Yes	Yes	Yes	Yes
Was the study apparently free of other problems that could put it at a high risk of bias?	Yes	Yes	Yes	Yes
